# Targeting Myeloid-Derived Suppressor Cells to Enhance the Antitumor Efficacy of Immune Checkpoint Blockade Therapy

**DOI:** 10.3389/fimmu.2021.754196

**Published:** 2021-12-22

**Authors:** Xueyan Li, Jiahui Zhong, Xue Deng, Xuan Guo, Yantong Lu, Juze Lin, Xuhui Huang, Changjun Wang

**Affiliations:** ^1^ School of Traditional Chinese Medicine, Southern Medical University, Guangzhou, China; ^2^ Guangdong Provincial People’s Hospital, Guangdong Academy of Medical Sciences, Guangdong Geriatric Institute, Guangzhou, China; ^3^ Guangzhou University of Chinese Medicine, Guangzhou, China; ^4^ School of Medicine, South China University of Technology, Guangzhou, China

**Keywords:** myeloid-derived suppressor cells (MDSCs), immune checkpoint blockade (ICB) therapy, immunosuppression, programmed cell death protein 1 (PD-1), the tumor microenvironment (TME)

## Abstract

Myeloid-derived suppressor cells (MDSCs) are a heterogeneous population of immature myeloid cells that are activated under pathological conditions, such as cancer, or mature myeloid cells that are converted immune-suppressive cells *via* tumor-derived exosomes, and potently support the tumor processes at different levels. Currently, multiple studies have demonstrated that MDSCs induce immune checkpoint blockade (ICB) therapy resistance through their contribution to the immunosuppressive network in the tumor microenvironment. In addition, non-immunosuppressive mechanisms of MDSCs such as promotion of angiogenesis and induction of cancer stem cells also exert a powerful role in tumor progression. Thus, MDSCs are potential therapeutic targets to enhance the antitumor efficacy of ICB therapy in cases of multiple cancers. This review focuses on the tumor-promoting mechanism of MDSCs and provides an overview of current strategies that target MDSCs with the objective of enhancing the antitumor efficacy of ICB therapy.

## 1 Introduction

Myeloid-derived suppressor cells (MDSCs) are broadly defined as a heterogeneous population of immature myeloid cells sharing many phenotypic markers with monocytes, macrophages, and dendritic cells (DCs), leading a powerful immunosuppressive role in the tumor microenvironment (TME). Notably, some studies have reported that normal monocytes could be converted to MDSC *via* tumor-derived exosomes (1); therefore, MDSCs represent not only immature but also mature myeloid cells. Indeed, also human PMN-MDSC do not primarily represent immature cells. Stephan Lang et al. demonstrated that a subset of mature CD11b+/CD16+ PMN-MDSC was identified as the MDSC subset with the strongest immunosuppressive activity and the highest clinical relevance (2). MDSCs represent an intrinsic part of the myeloid-cell lineage and are comprised of myeloid-cell progenitors and precursors of myeloid cells. In normal physiological states, MDSCs quickly differentiate into mature granulocytes, macrophages, or DCs. However, in pathological conditions such as cancer, MDSCs are abnormally activated and exhibit potent immune-suppressive activity (3).

Immune checkpoint blockade (ICB) therapy has emerged as the standard therapy for the treatment of cancer due to its unprecedented and durable responses in patients with refractory cancers (4). Induction of immune checkpoints, such as cytotoxic T lymphocyte antigen 4 (CTLA-4), programmed cell death protein 1 (PD-1) and its ligand, PD-L1, mediates tumor immune evasion. CTLA-4 competes with the co-stimulatory receptor CD28 for binding to B7 ligands. PD-1 is expressed by activated T cells, while its ligands PD-L1 and PD-L2 are expressed by tumor and immune cells. The PD-1 pathway is important for driving T cells into a dysfunctional state known as T cell exhaustion. ICB employs antibody-based therapies targeting these checkpoints in an effort to unleash preexisting adaptive immunity (5). However, it is clear from large clinical trials that therapeutic resistance occurs in numbers of patients leading to ultimately progression (6–8). Extensive preclinical researches indicated that targeting MDSCs could be a promising strategy to lead the TME reprogramming and enhance the antitumor efficacy in combination with ICB therapy. In this review, we discuss the classification and protumoral mechanisms of MDSCs, including immunosuppressive functions and non-immunosuppressive mechanisms. We summarize therapeutic strategies targeting them to enhance the antitumor activity of ICB therapy.

## 2 Classification of MDSCs

MDSCs are mainly divided into granulocytic/polymorphonuclear MDSCs (G/PMN-MDSCs) and monocytic MDSCs (M-MDSCs), according to their origin from the granulocytic or monocytic myeloid cell lineages, respectively. In mice, MDSCs are broadly identified as CD11b+Gr1+ cells. Based on the variable expression of the Gr-1 marker, M-MDSCs are defined as CD11b+Ly6ChiLy6G−; conversely, PMN-MDSCs are defined as CD11b+Ly6CloLy6G+. In humans, M-MDSCs are characterized as CD11b+CD33+HLA-DR−/lowCD14+CD15−, whereas PMN-MDSCs are phenotypically CD11b+CD33+HLA-DR−/lowCD14-CD15+(CD66b+) (9). A small group of myeloid precursor cells with MDSCs features has also been identified in humans (but not in mice) and named “early MDSCs.” This group of cells with potent immunosuppressive features, defined as HLA-DR−CD33+Lin−(CD3-CD14-CD15-CD19-CD56-) and represents less than 5% of the total population of MDSCs (10). An important issue in this viewpoint is the great heterogeneity of these cells, which makes the identification and isolation of human MDSC subsets very challenging. However, it is important to bear in mind that the potent immune-suppressive activity of MDSCs is the most reliable marker distinguishing MDSCs from mature neutrophils and monocytes.

## 3 The Tumor-Promoting Mechanism of MDSCs

### 3.1 The Contribution of MDSCs to the Immune Suppressor Network in the TME

**Figure 1:**
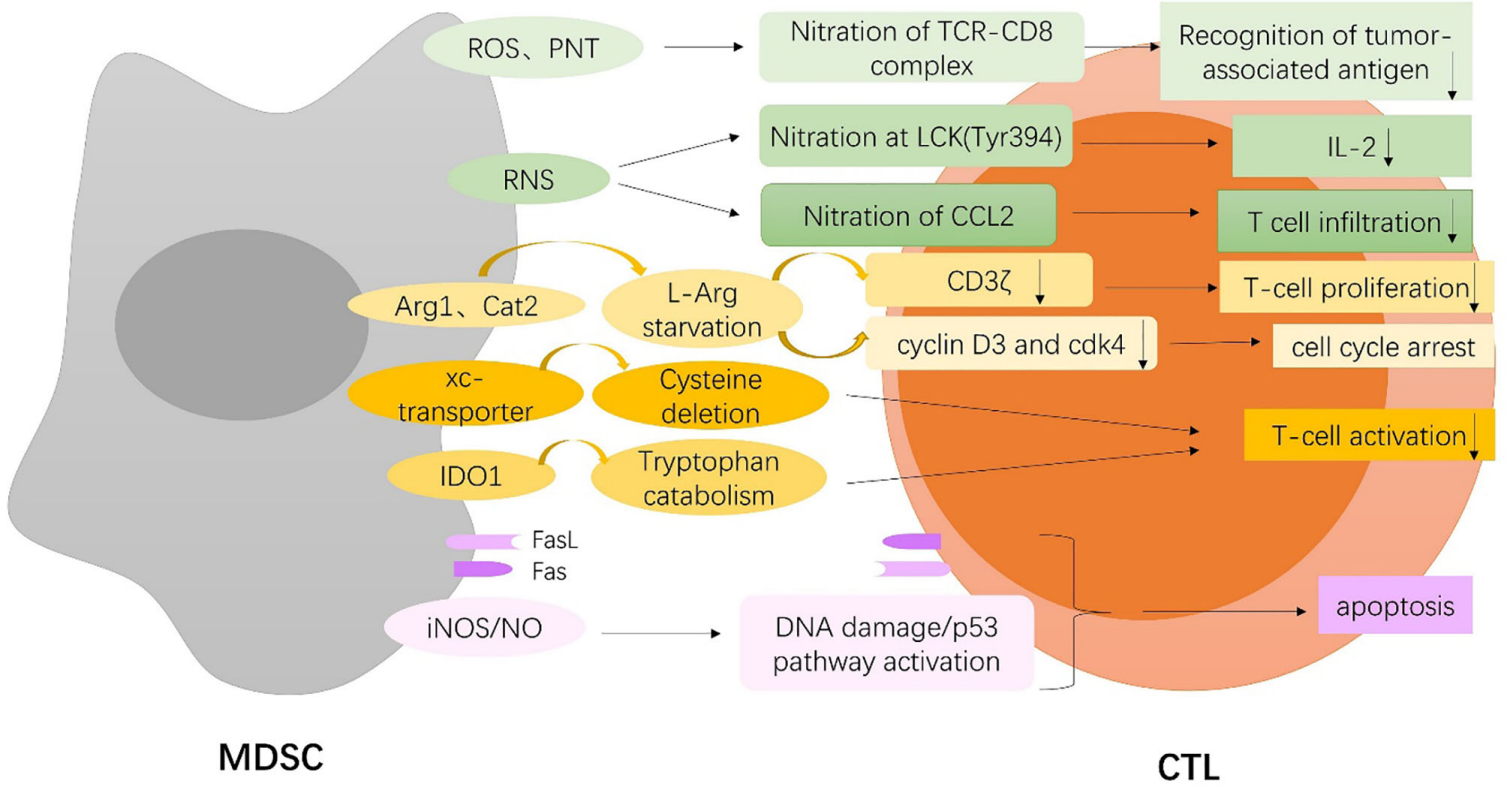
MDSCs-mediated inhibition of T cell. MDSCs induce nitration of TCR-CD8 complex through hyperproduction of reactive oxygen species (ROS) and peroxynitrite (PNT) and nitrate lymphocyte-specific protein tyrosine kinase (LCK) at Tyr394 and CCL2 chemokine through reactive nitrogen species (RNS), which inhibit T cell recognition of tumor antigen, and lead to reduced interleukin 2 (IL2) production and T-cell infiltration. MDSCs deplete extracellular L-arginine (L-Arg) by expressing arginase-1(Arg-1) and cationic amino acid transporter 2 (Cat2), which block the re-expression of CD3zeta, inhibit antigen-specific T-cell proliferation, and result in cell cycle arrest through upregulation of cyclin D3 and cyclin-dependent kinase 4 (cdk4). MDSCs also limit the availability of cysteine and tryptophan by expressing the xc-transporter and IDO, respectively, which block T-cell activation. On the one hand, MDSCs trigger apoptosis of tumor-infiltrating CTLs through the Fas/Fas-ligand axis. On the other hand, MDSCs express the death receptor Fas and apoptose in response to T-cell-expressed FasL. MDSCs induce DNA damage and subsequent p53 pathway activation through an iNOS-dependent pathway, thus inducing apoptosis of CD8+ T cells.

**Figure 2:**
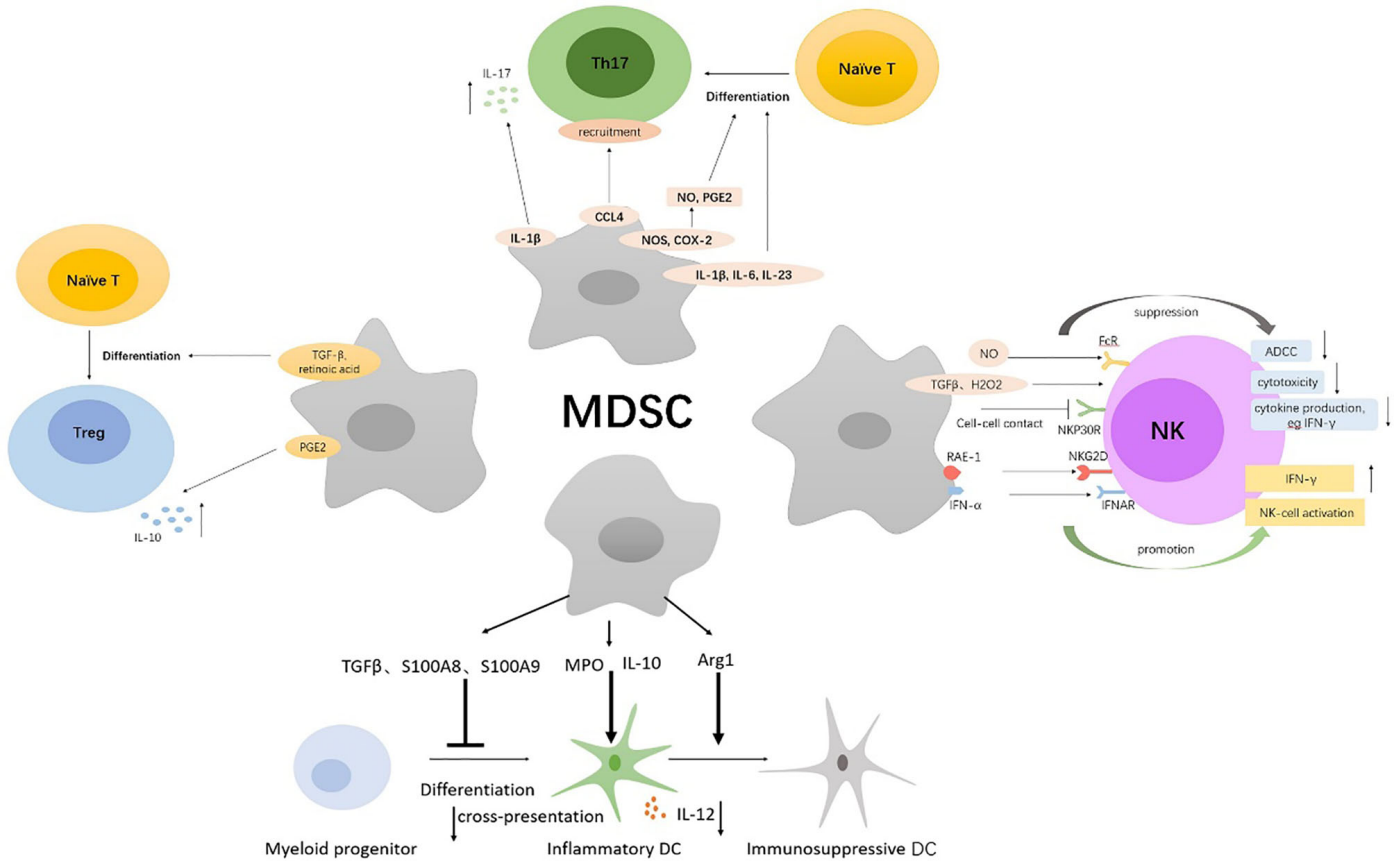
Multiple immunosuppressive mechanisms of MDSCs on NK, DCs, Th17, Tregs. MDSCs directly inhibit autologous natural killer (NK) cell cytotoxicity and cytokine secretion by interaction with the NKp30 on NK cells. Also, MDSCs indirectly inhibit NK-cell FcR-mediated functions including antibody-dependent cellular cytotoxicity (ADCC), cytokine production, and signal transduction through the production of NO, TGFβ, and H2O2. Immature NK cells can be converted into MDSCs by tumor-derived GM-CSF. MDSCs activate NK cells to produce high amounts of IFN‐γ, which depends partially on the interaction of NKG2D on NK cells with NKG2D ligand RAE-1 on MDSCs and the IFNAR pathway. MDSCs inhibited IL-12 production of DCs by IL-10 and suppressed T-cell stimulatory activity of DCs. Myeloperoxidase (MPO)-driven lipid peroxidation in PMN-MDSCs blocked cross-presentation by DCs. Arg1-dependent production of polyamines by MDSCs conditioned DCs toward an immunosuppressive phenotype *via* activation of the Src kinase. S100A8 and S100A9 produced by MDSCs inhibited DCs differentiation from hematopoietic progenitor cells (HPCs) *via* persistent upregulation of ROS. MDSCs secrete Th17-driving cytokines (IL-1β, IL-6, and IL-23) and produce NO and Prostaglandin-E2 (PGE2) to facilitate Th17 cells differentiation; the latter required nitric oxide synthase (NOS) and cyclooxygenase 2 (COX-2) activity. Additionally, MDSCs promote the recruitment of Th17 cells through CCL4 and induce secretion of IL-17 by CD4(+) T cells through secretion of IL-1β. MDSCs induce Foxp3+ Tregs from naive CD4+ T cells and monocyte-induced Th17 cells *via* MDSCs-derived TGF-β and retinoic acid. PGE2 produced by MDSCs expand IL-10-producing Treg subsets.

MDSCs significantly inhibit the antitumor activity of T cells, especially cytotoxic T lymphocytes (CTLs) (**Figure 1**), and also make pro-inflammatory cells, such as NK cells and DCs, incompetent; in addition, MDSCs induce the generation of Tregs and Th17 cells, which remodel the microenvironment that supports tumor development (**Figure 2**).

#### 3.1.1 MDSCs and CTLs

Firstly, MDSCs directly disrupt the binding of specific peptide-major histocompatibility complex (pMHC) dimers to CD8-expressing T cells through nitration of tyrosines in a T-cell receptor (TCR)-CD8 complex, which makes CD8+ T cells unable to bind pMHC and to respond to the specific peptide, although they retain their ability to respond to non-specific stimulation. Nitration of TCR-CD8 is induced by MDSCs through hyperproduction of reactive oxygen species (ROS) and peroxynitrite (PNT) during direct cell-cell contact (11). More specifically, Shan Feng et al. identified that lymphocyte-specific protein tyrosine kinase (LCK), an initiating tyrosine kinase in the T-cell receptor signaling cascade, was nitrated at Tyr394 by MDSCs through reactive nitrogen species (RNS), which inhibited T-cell activation, leading to reduced interleukin 2 (IL2) production and proliferation (12). Additionally, MDSCs can directly act on tumor cells to interfere with the recognition of tumor antigens by T cells. Increased level of free radical PNT by MDSCs at the tumor site induces post-translational modifications of cell surface molecules on tumor cells and inhibits binding of processed peptides to tumor cell-associated MHC, as a result, rendering them resistant to antigen-specific CTLs (13). Also, intratumoral RNS production by MDSCs induces CCL2 chemokine nitration and hinders T-cell infiltration, resulting in the trapping of tumor-specific T cells in the stroma that surrounds cancer cells (14). Notably, MDSCs do not block early steps of T-cell activation for that T cells express multiple early activation markers even in the presence of MDSCs, but rather induce DNA damage and subsequent p53 pathway activation in CD8+ T cells through an inducible nitric oxide synthase(iNOS)-dependent pathway, thus inhibiting proliferation and inducing apoptosis of CD8+ T cells even in the presence of DCs presenting a high-affinity cognate peptide (15). Similarly, MDSCs potently block proliferation of T cells stimulated with either mitogen or antigenic peptide by a nitric oxide (NO)-dependent mechanism without resulting in an inability to upmodulate the early activation markers, which characterizes the distinctive capacity of MDSCs to generate suppressive signals only when encountering activated T cells (16).

MDSCs are also found to express high levels of arginase-1(Arg-1) and upregulate cationic amino acid transporter 2 (Cat2), which allow them to rapidly deplete extracellular L-arginine (L-Arg) and thereby block the re-expression of CD3zeta in stimulated T cells and inhibit antigen-specific T-cell proliferation (17, 18). Also, L-Arg starvation results in cell cycle arrest, which is associated with the inability of T cells to upregulate cyclin D3 and cyclin-dependent kinase 4 (cdk4) (19). However, it is worth noting that a preclinical study indicates that Arg-1 is neither constitutively expressed in MDSCs nor required for MDSC-mediated inhibition of T-cell proliferation, which is rather dependent on direct cell contacts undiminished by PD-L1 blockade (20). MDSCs also block T-cell activation by sequestering cystine and limiting the availability of cysteine, which is an essential amino acid for T-cell activation, because T cells lack cystathionase, which converts methionine to cysteine, and because they do not have an intact xc-transporter and therefore cannot import cystine and reduce it intracellularly to cysteine. T cells depend on antigen-presenting cells (APC), such as macrophages and DCs, to export cysteine, which is imported by T cells *via* their ASC neutral amino acid transporter. MDSCs express the xc-transporter and import cystine; however, they do not express the ASC transporter and do not export cysteine. MDSCs compete with APC for extracellular cystine, and in the presence of MDSCs, APC release of cysteine is reduced, thereby limiting the extracellular pool of cysteine (21). In addition, indoleamine 2,3-dioxygenase (IDO), produced by MDSCs, catabolizes the amino acid tryptophan, resulting in GCN2 kinase-mediated proliferative arrest and anergy induction in T cells (22).

Finally, it has been reported that PMN-MDSCs expressing high levels of Fas-ligand trigger apoptosis of tumor-infiltrating lymphocytes through the Fas/Fas-ligand axis, which results in immunotherapy resistance in the autochthonous TiRP melanoma model (23). Moreover, MDSCs are shown to express the death receptor CD95 and induce T-cell apoptosis *via* CD95 ligand expressed on activated T cells (24). Interestingly, MDSCs also express the death receptor Fas and apoptose in response to T cell-expressed FasL, which shows a retaliatory relationship between T cells and MDSCs in that MDSCs suppress T-cell activation; however, once activated, T cells mediate MDSCs apoptosis (25).

#### 3.1.2 MDSCs and NK

MDSCs have been shown to directly inhibit autologous natural killer (NK) cell cytotoxicity and cytokine secretion through cell contact, which is dependent mainly on the NKp30 on NK cells (26). Julien Cherfils-Vicini (27) and Tobias Eggert (28) also confirmed that in melanoma and HCC, recruitment of MDSCs in tumor site enhanced the inhibition of NK cell functionality, manifested in significantly decreased NK cell degranulation and IFN-γ production, and strongly affected NK cell cytotoxicity, ultimately resulting in tumor progression and metastasis. Additionally, MDSCs from patients with cancer were found to obviously inhibit NK-cell FcR-mediated functions including antibody-dependent cellular cytotoxicity (ADCC), cytokine production, and signal transduction in a contact-independent manner, in part through NO production (29). Both M-MDSCs and G-MDSCs inhibited NK-cell activity through the production of TGFβ, while PMN-MDSCs also suppressed NK-cell function through the production of H2O2 (30, 31). What is more, in the TME, immature NK cells can be converted into MDSCs by tumor-derived granulocyte-macrophage colony-stimulating factor (GM-CSF) to assist tumor cells to escape immune surveillance (32). As revenge, NK cells can also directly kill MDSCs. CD8+NKT-like cells expressing both T-cell activation markers and NK cell markers clear tumor antigen-bearing MDSCs to improve the antitumor microenvironment in a granzyme B-dependent manner (33). Paradoxically, MDSCs can activate NK to some extent. Mononuclear Gr-1(+) CD11b (+) F4/80(+) MDSCs isolated from RMA-S tumor-bearing mice did not suppress but activated NK cells to produce high amounts of IFN‐γ, and NK-cell activation by MDSCs depended partially on the interaction of NKG2D on NK cells with NKG2D ligand RAE-1 on MDSCs (34). Moreover, MDSCs produced IFN-α after polyinosinic: polycytidylic acid treatment and activated NK cells through the IFNAR pathway, conferring tumor-suppressive functions on NK cells (35).

#### 3.1.3 MDSCs and DCs

Dendritic cells (DCs) are a critical component of immune responses in cancer. Firstly, MDSCs weaken the pro-inflammatory and antigen-presenting functions of DCs. It has been reported that MDSCs inhibited TLR ligand-induced IL-12 production of DCs by IL-10 production and suppressed T-cell stimulatory activity of DCs, thus impairing dendritic cell function and promoting tumor development (36). Additionally, myeloperoxidase (MPO)-driven lipid peroxidation in PMN-MDSCs blocked cross-presentation by DCs without affecting the direct presentation of antigens by these cells, which did not require direct cell-cell contact and was associated with the transfer of lipids. Pharmacological inhibition of MPO in combination with checkpoint blockade reduced tumor progression in different tumor models (37). Secondly, MDSCs can induce the transformation of pro-inflammatory DCs into immunosuppressive DCs. For example, Arg1-dependent production of polyamines by MDSCs conditioned DCs toward an IDO1-dependent, immunosuppressive phenotype *via* activation of the Src kinase, which has IDO1-phosphorylating activity (38). Finally, various tumor-derived factors induce DCs to convert to MDSCs. Upregulation of the Inhibitor of Differentiation 1 (ID1), in response to tumor-secreted factors, such as TGFβ, is responsible for the switch from DCs differentiation to MDSC expansion during tumor progression and metastasis (39). Tumor-induced upregulation of the myeloid-related protein S100A8 and S100A9, which also can be produced by MDSCs, inhibit DCs differentiation from hematopoietic progenitor cells (HPCs) *via* persistent upregulation of ROS in progenitor cells and induced accumulation of MDSCs (40).

#### 3.1.4 MDSCs and Th17

The development of cancer has been linked to chronic inflammation. IL-17–secreting CD4+ T cells, namely, T-helper 17 (Th17) cells, are a kind of important inflammatory component and have been found to promote the frequency of certain tumors. The mechanisms of MDSCs regulating Th17 mainly involve (1) induction of Th17 differentiation, (2) promotion of Th17 recruitment, (3) facilitation of IL-17 secretion. It has been demonstrated that MDSCs not only secrete Th17-driving cytokines (IL-1β, IL-6, and IL-23) but also produce NO and Prostaglandin-E2 (PGE2) to facilitate Th17 cells differentiation, which required nitric oxide synthase (NOS) and cyclooxygenase 2 (COX-2) activity, consequently portraying chronic inflammatory state to facilitate tumor growth in oral squamous cell carcinoma and ovarian cancer (41, 42). IDO1 expression in MDSCs was also found to play a role in regulating the polarization of Th1, Th17, and possibly T regulatory cells in pancreatic adenocarcinoma (43). In addition, MDSCs have been reported to directly contribute to tumor formation by CCL4-mediated recruitment of Th17 cells (44). Interleukin-17 (IL-17) is a signature cytokine of Th17 cells. Chemotherapy-triggered IL-1β secretion by MDSCs induced secretion of IL-17 by CD4(+) T cells, which blunted the anticancer efficacy of the chemotherapy (45). Meantime, IL-17 also increased the infiltration of MDSCs in tumors to promote tumor development (46). However, another study found that in breast cancer, IL-17 significantly induced MDSCs differentiation, inhibited their proliferation, and triggered apoptosis *via* activating Stat3, although low IL-17 inhibited the activation of Stat3, leading to increase formation of MDSCs (47). Thus, the role of IL-17 in the regulation of MDSCs needs to be further explored, which may depend on tumor types and IL-17 concentrations.

#### 3.1.5 MDSCs and Tregs

Regulatory T cells (Tregs), another immunosuppressive cell population, play an important role in the control of inflammatory responses as well as in the suppression of antitumor immune responses. It has been reported that in pancreatic ductal adenocarcinoma (PDCA), MDSCs can induce Treg cells in a cell-cell dependent manner; meanwhile, Treg cells affect the survival and/or the proliferation of MDSCs (48). MDSCs are also found to induce Foxp3+ Tregs when cocultured with naive CD4+ T cells and catalyze the transdifferentiation of Foxp3+ regulatory T cells from monocyte-induced Th17 cells, which is dependent on MDSCs-derived TGF-β and retinoic acid (49). PGE2, another immunosuppressive factor from MDSCs, potentiates the suppressive functions of M-MDSCs and increases their capacity to expand IL-10-producing Treg subsets but lowers their capacity to induce TGF-β-producing Tregs (50). The interactions between MDSCs and Treg cells further contribute to the immunosuppressive environment.

In addition to Treg cell stimulation, MDSCs could shift macrophages to an M2-like phenotype with immuno-suppressive features and low IL-12 production, thereby promoting tumor growth. MDSCs are also reported to block lymphocyte homing by downregulating the cell adhesion molecule L-selectin on CD4+and CD8+T cells and impair the extravasation and tissue infiltration of T cells through the downregulation of CD44, a receptor for the extracellular matrix component hyaluronic acid (HA), and CD162, a selectin P ligand (51).

### 3.2 Non-Immunosuppressive Mechanisms of MDSCs

#### 3.2.1 Promoting Angiogenesis

**Figure 3:**
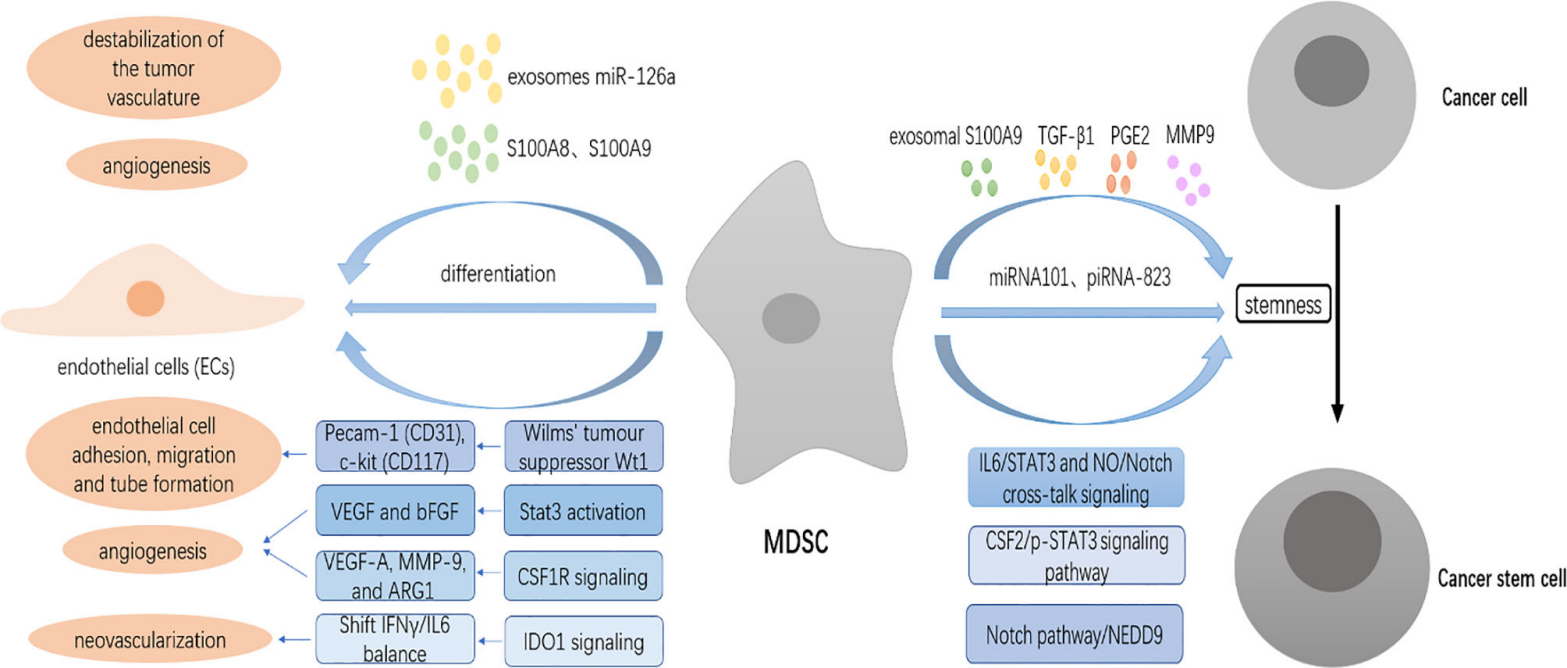
MDSCs promote angiogenesis and induce cancer stem cells (CSCs) in the TME. MDSCs contribute to tumor vascularization by producing high levels of matrix metalloproteinase 9 (MMP9). MDSCs directly produce angiogenic factors, including VEGF and bFGF through Stat3 activation to induce angiogenesis. MDSCs control the expression of CD31 and CD117 through the expression of the Wilms’ tumor suppressor Wt1, causing tumor vascularization. MDSCs shift the IFNγ/IL6 balance to promote neovascularization through IDO1 signaling. MDSCs-secreted S100A8 and S100A9 stimulate angiogenesis. Exosomes miR-126a released from MDSCs promote tumor angiogenesis. MDSCs differentiate into endothelial cells (ECs). MDSCs endow stem-like qualities to breast cancer cells through IL6/STAT3 and NO/Notch cross-talk signaling and to epithelial ovarian cancer (EOC) cells by the colony-stimulating factor 2 (CSF2)/p-STAT3 signaling pathway. MDSCs also trigger the expression of miRNA101 and piRNA-823 to promote the stemness of ovarian carcinoma cells and myeloma (MM) cells, respectively. MDSCs can indirectly modulate the stemness of tumor cells through the secretion of various factors such as exosomal S100A9, TGF-β1, PGE2, MMP9, and chitinase 3-like 1 (CHI3L1).

In addition to suppressing host immune functions within the TME, MDSCs also promote angiogenesis, a step essential for tumor growth and progression, which indicates that another possible mechanism to enhance antitumor activity when combined with ICB therapy and targeting MDSCs. A large number of studies have shown that the accumulation of MDSCs is highly correlated with angiogenesis in tumors. Prevention of MDSCs accumulation through stem-cell factor (SCF) silencing in tumor cells could result in reduced blood vessel formation in the tumor site (52). ETS transcription factor ELF5-mediated recruitment of MDSCs drives vasculogenesis and lung metastasis in luminal breast cancer (53). In hepatocellular carcinoma, tumor blood vessel density increased when HCC cells and tumor‐associated MDSCs (T‐MDSCs) were subcutaneously injected into mice compared with HCC cells alone (54). More specifically, MDSCs can regulate angiogenesis through multiple mechanisms (**Figure 3**). Firstly, MDSCs contribute to tumor vascularization by producing high levels of matrix metalloproteinase 9 (MMP9), a critical regulator of tumor angiogenesis and vasculogenesis, which releases vascular endothelial growth factor (VEGF) from the matrix (55). Also, MDSCs directly produce angiogenic factors, including VEGF and bFGF, through Stat3 activation to induce angiogenesis, and Stat3-regulated factors produced by MDSCs also induce constitutive activation of Stat3 in tumor endothelium, which is required for tumor factor-induced endothelial migration and tube formation (56). In melanoma and lung and prostate tumors, targeting recruitment of MDSCs by inhibiting CSF-1 receptor inhibits tumor angiogenesis associated with reduced expression of proangiogenic genes such as VEGF-A and MMP-9 and reverses tumor resistance to antiangiogenic therapy (57).

Pecam-1 (CD31), an endothelial cell-cell adhesion molecule, is important in the formation of new vessels (58). C-kit (CD117) is expressed by human and rodent endothelial cells, and it has been shown that stem cell factor, a c-kit ligand, stimulates migration and tube formation of human endothelial cells. MDSCs have been reported to transcriptionally control the expression of CD31 and CD117 through the frequent expression of the Wilms’ tumor suppressor Wt1, and knockout of Wt1 in MDSCs is sufficient to cause regression of tumor vascularization (59). In addition, IFNγ and IL6 are two inflammatory cytokines that appear to work at cross-purposes with regard to restraining and promoting angiogenesis, respectively. A subpopulation of MDSCs designated as IDVCs (IDO1-dependent vascularizing cells) shifts the IFNγ/IL6 balance to promote neovascularization through IDO1 signaling (60). MDSCs-secreted S100A8, which acts by destabilization of the tumor vasculature, represents a resistance-conferring factor induced by antiangiogenic therapies (AAT), which can be reverted by combining with ATRA through reduction of MDSCs levels (61). S100A9, mainly produced by MDSCs, is also able to stimulate angiogenesis (62). A growing number of studies demonstrate that exosomes released by MDSCs play a critical role in promoting tumor effect. It has recently been shown that exosomes miR-126a released from MDSCs promote tumor angiogenesis resulting in breast tumor lung metastasis (63).

Finally, MDSCs are also found to differentiate into endothelial cells (ECs) and acquire EC properties in the TME (55). Expression of kinase insert domain receptor (KDR), also known as VEGFR2, considered a specific marker for endothelial progenitors within BMDCs, is responsible for MDSCs of differentiation from hematopoietic progenitor cells (HPCs) and proangiogenic function, the latter required for the angiogenic switch necessary for malignant progression of low-grade to high-grade tumors, which indicate that VEGFA may be a potential target of MDSCs and enhancing antitumor activity when combined with ICB (64).

#### 3.2.2 Induction of Cancer Stem Cells

Another nonimmunologic mechanism through which MDSCs drive tumor progression is the induction of cancer stem cells (CSCs). CSCs, which are also known as tumor-initiating cells and stem-like cancer cells, are a subset of tumor cells associated with tumor progression and treatment resistance that possess characteristics associated with normal stem cells, specifically the ability to self‐renew and differentiate into multiple cell types. Although the phenotype of CSCs has not arrived at consensus due to large heterogeneity, several markers such as side population (SP), sphere formation capacity, and CSCs core genes have been used to identify CSCs (65). MDSCs increase cancer cell stemness *via* distinct mechanisms in many cancer types (**Figure 3**). Recently, MDSCs have been shown to promote the stemness of esophageal squamous cell carcinoma (ESCC) cells through neural precursor cell expressed, developmentally downregulated 9 (NEDD9) that is required to maintain the stem-like phenotype *via* the Notch pathway, and in turn, NEDD9 regulates CXCL8 through the ERK pathway to recruit MDSCs into the tumor (66). MDSCs are also reported to endow stem-like qualities to breast cancer cells through IL6/STAT3 and NO/Notch cross-talk signaling (67). Xiaofeng Li et al. confirmed that MDSCs dramatically promoted tumor sphere formation, cell colony formation, and CSC accumulation, and enhanced the expression of the stemness biomarkers NANOG and c-MYC in epithelial ovarian cancer (EOC) cells by inducing the colony-stimulating factor 2 (CSF2)/p-STAT3 signaling pathway (68).

Additionally, MDSCs modulate levels of non-coding RNAs whose expression and function are linked to cancer development and progression to promote the stemness of cancer cells. MDSCs triggered expression of miRNA101, a conserved non-coding RNA that fine-tunes gene expression, regulates cell differentiation and cell-fate determination, in ovarian carcinoma cells, which subsequently repressed the corepressor gene C-terminal binding protein-2 (CtBP2) that directly targeted stem cell core genes, thereby resulting in enhancing CSC gene expression, sphere formation, and increasing metastatic and tumorigenic potential (69). piRNA-823, another small non-coding RNA (ncRNA) participating in multiple myeloma (MM) proliferation, induced by G-MDSCs, has been demonstrated to endow stem-like qualities to MM cells (70).

Finally, MDSCs can indirectly modulate the stemness of tumor cells through the secretion of various factors. G-MDSCs have been shown to induce the stemness of colorectal cancer (CRC) cells that foster the development of CRC through exosomal S100A9, which can be enhanced by hypoxia in a hypoxia-inducible factor 1α (HIF-1α)-dependent manner (71). TGF-β1 produced by MDSCs is also found to increase cancer stem cells in A549 transplantation tumors (72). PGE2 derived from MDSCs conveys the stem cell-like properties to EOC cells (73). MDSCs secrete pro-metastatic factors such as MMP9 and chitinase 3-like 1 (CHI3L1) to promote triple-negative breast cancer (TNBC) stem cell function (74). Conversely, CSCs are also able to recruit MDSCs to regulate immunosuppression in the TME. For example, downregulation of microRNA-92 expression in CD133+ melanoma stem cells recruits MDSCs *via* enhancing integrin-dependent activation of TGFβ (75). Collectively, targeting cross-talk between MDSCs and CSCs could offer a unique locus to improve cancer treatment by coordinately targeting a coupled mechanism that enables cancer stemness and immune escape.

## 4 Therapeutic Targeting of MDSCs to Improve the Efficiency of ICB

It is now becoming increasingly evident that immune suppression may be centrally implicated in dictating resistance to ICB therapy in tumor treatment, and as MDSCs are one of the main immunosuppressive factors in cancer, therapeutic targeting of these cells could improve response rates and survival of patients with cancer. This section provides an overview of several different therapeutic strategies that are currently being developed to target MDSCs to enhance the antitumor effect of ICB therapy (**Table 1**).

**Table 1:** Therapeutic targeting of MDSCs to improve the efficiency of ICB.

Strategy	Target	Tumor types	Immunotherapy	Setting	References
Blockade of recruitment	CCL5	Melanoma and NSCLC	Anti-PD-1	Preclinical	(72)
	CXCL3	Colorectal cancer (CRC)	Anti-PD-1	Preclinical	(73)
	CXCR4	Osteosarcoma, glioblastoma, and ovarian cancer	Anti-PD-1	Preclinical	(74–76)
	CCR2	Gliomas and CRC	Anti-PD-1	Preclinical	(77, 78)
	IL-6	Prostate cancer	Anti-PD-1 and anti-CTLA-4	Preclinical	(79)
	CSF-1R	Breast cancer, colon cancer, and melanoma	Anti-CTLA-4	Preclinical	(80)
	CSF-1R	Neuroblastoma, melanoma, and colon cancer	Anti-PD-1 or/and anti-CTLA-4	Preclinical	(81, 82)
	CSF1	Triple-negative breast cancer (TNBC) and CRC	Anti-PD-1	Preclinical	(83)
	Lactate	Melanoma and CRC	Anti-PD-1	Preclinical	(84)
Inhibition of expansion	NLRP3/IL-1β signaling	Melanoma, PDAC, LLC, and renal cancer	Anti-PD-1	Preclinical	(85–88)
	NLRP3/IL-18 signaling	Lymphoma	Anti-PD-1	Preclinical	(89)
	VEGF or TGF-β	Mesothelioma (MPM), LLC and melanoma	Anti-CTLA-4 or/and anti-PD-1	Preclinical	(90–92)
	CD200R	PDAC	Anti-PD-1	Preclinical	(92)
Promotion of differentiation	Prostaglandin E2 (PGE2) receptor 4 (EP4)	CRC, mammary carcinoma, fibrosarcoma, and PDAC	Anti-PD-1 or anti-CTLA-4	Preclinical	(93–95)
	Polyamine	Melanoma and mammary carcinoma	Anti-PD-1	Preclinical	(96)
	All-trans retinoic acid (ATRA)	Colon cancer and breast cancer	Anti-PD-1	Preclinical	(97)
	ATRA	Melanoma	Anti-PD-1	clinical	(98)
	HMGB1	Breast cancer and NSCLC	Anti-PD-1	Preclinical	(99)
Inhibition of immunosuppressive function	NOX2	Lymphoma and CRC	Anti-PD-1	Preclinical	(100)
	Aurora A	Breast cancer	Anti-PD-L1	Preclinical	(101)
	Poly (ADP-ribose) polymerase (PARP)	Colon cancer	Anti-PD-1	Preclinical	(102)
	PARP	mCRPC	Anti-PD-1	clinical	(103)
	Histone deacetylase	Breast cancer, metastatic pancreatic cancer, lymphoma, melanoma, lung, and renal cell carcinoma	Anti-PD-1 and anti-CTLA-4	Preclinical	(104–106)
	Phosphoinositide 3-kinase (PI3K)	Osteosarcoma, colon, and oral cancer	Anti-PD-L1	Preclinical	(107–109)
	Myeloid cell receptor tyrosine kinases	Melanoma	Anti-PD-1	Preclinical	(110)
	Semaphorin4D	Oral cancer	Anti-PD-1 or anti-CTLA-4	Preclinical	(111)
	TGF-β	Esophageal squamous cell carcinoma (ESCC)	Anti-PD-1	Preclinical	(112)
	FATP2	LLC and melanoma	Anti-PD-1 or anti-CTLA-4	Preclinical	(113)

### 4.1 Targeting the Recruitment of MDSCs

MDSCs are actively recruited to primary and metastatic tumor sites, which are regulated by chemokines with little specificity in the types of chemokines produced by different tumors, and blocking the interactions with their ligands is a rational approach to inhibit MDSCs accumulation in the TME. In particular, therapeutic blockade of CCL5-CXCR2 interaction by disrupting production of CCL5 or CXCR2 antagonist has demonstrated promising antitumor efficacies in several preclinical cancer models. For example, a non-canonical Wnt ligand-YAP signaling axis regulated the recruitment of PMN-MDSCs to the tumor bed by promoting the expression of CXCR2-dependent chemokines CCL5 in response to PD-1 blockade. Pharmacologic inhibition of Wnt ligand signaling supports anti-PD-1 efficacy by reversing the recruitment of G-MDSCs in an autochthonous model of melanoma and non-small-cell lung cancer (NSCLC) (76). CXCL3, another CXCR2-dependent chemokine, is implicated in the recruitment of MDSCs to the tumor. In KRAS-mutant colorectal cancer (CRC), KRAS∗-mediated repression of interferon regulatory factor 2 (IRF2) that directly represses CXCL3 expression results in high expression of CXCL3, which binds to CXCR2 on MDSCs and promotes their migration to the TME. Anti-PD-1 resistance of KRAS∗-expressing tumors can be overcome by enforced IRF2 expression or by inhibition of CXCR2 (77). The inhibition of CXCR4 also leads to a reduction of MDSCs infiltration. In osteosarcoma, CXCR4+MDSCs migrate tumor tissues toward an SDF-1 gradient, which could inhibit CTL expansion and result in resistance to ICB. AMD3100, a highly specific CXCR4 antagonist, has a synergistic effect with anti-PD-1 antibody in tumor treatment (78). Similarly, in glioblastoma and ovarian cancer, combination therapy of anti-CXCR4 and anti-PD-1 is also demonstrated to decrease populations of immunosuppressive tumor-infiltrating MDSCs, improve CD4+/CD8+ ratios, and confer a significant survival benefit compared to control and monotherapy arms (79, 114). The CCL2–CCR2 axis has also a critical role in tumor progression since it supports tumor invasion and migration of MDSCs to the tumor site. CCR2 inhibition decreases tumor-associated MDSCs and thus unmasks an anti-PD-1 survival benefit to slow the progression of resistant murine gliomas (80). In CRC mouse models, incomplete radiofrequency ablation(iRFA) induces sustained infiltration of MDSCs in residual tumors through tumor cell-derived CCL2, which inhibits T-cell function and hinders the efficacy of anti-PD-1 therapy. Administration of a CCR2 antagonist or the loss of CCL2 expression in tumor cells enhances antitumor immunity in the residual tumor and overcomes the resistance to ICB therapy (81). In BRAFV600E inhibitors (BRAFi)-resistant melanomas, the addition of MDSC depletion/blockade (anti-Gr-1 + CCR2 antagonist) prevented outgrowth of BRAFi-resistant tumors, although combination checkpoint blockade (anti-CTLA-4 + anti-PD-1) was ineffective (82).

It has been demonstrated that IL-6 is involved in MDSCs infiltration of tumors leading to increased tumor progression. Targeting of IL-6 directly or indirectly has therefore emerged as a strategy to limit the attraction of MDSCs or block their tumor-promoting functions. In PTEN-deficient prostate cancer, prostate-specific deletion of CHD1 resulted in markedly delayed tumor progression and prolonged survival, which was associated with a reduction in MDSCs and an increase in CD8+ T cells through IL-6, a key transcriptional target of CHD1. Pharmacologic inhibition of IL6 in combination with ICB elicits robust antitumor responses in prostate cancer (83).

Another well-characterized target to reduce MDSCs trafficking is the colony-stimulating factor 1 receptor (CSF1R) whose expression is restricted to monocytes and macrophages. CSF-1 signaling through its receptor CSF-1R is a critical regulator of survival, differentiation, and proliferation of myeloid cells and their precursors. In breast cancer, colon cancer, and melanoma, inhibition of CSF-1/CSF-1R signaling using an anti-CSF-1R antibody can regulate both the number and the function of MDSCs, induce antitumor T-cell responses and tumor regression when combined with CTLA-4 blockade therapy (115). In neuroblastoma, M-CSF/CSF-1R interaction interferes with the early development of myeloid cells and enables suppressive functions on human monocytes. Antagonizing CSF-1R with a selective inhibitor (BLZ945) modulates the induction of human and murine suppressive MDSCs and efficiently limit tumor progression. While checkpoint inhibitors are insufficient in controlling tumor growth, combining BLZ945 with PD-1/PD-L1 blocking antibodies results in superior tumor control (84). In IDO-expressing melanoma and colon cancer, inhibition of CSF-1R signaling can functionally block tumor-infiltrating MDSCs and enhance antitumor T-cell responses, thus sensitizing IDO-expressing tumors to immunotherapy with T-cell checkpoint blockade (85). In addition, estrogen receptor beta (ERβ) agonist LY500307 reduced tumor-derived CSF1 and decreased infiltration of CSF1R+ MDSCs in the tumor bed. A combined treatment of LY500307 and PD-1 antibody improved therapeutic efficacy in mouse tumor models, compared with monotherapies (86). However, in muscle-invasive bladder cancer (MIBC), GM-CSF significantly decreased the accumulation of MDSCs in both the blood and TME. Supplementary GM-CSF to neoadjuvant gemcitabine and cisplatin (GC) plus PD-L1 blockade could decrease local recurrence (LR) after radical surgery by immune modulation in the blood and TME (87). Therefore, it is undetermined that targeting CSF/CSFR signaling combined with ICB enhances antitumor efficacy, which may depend on tumor types. Finally, the recruitment of MDSCs to the tumor sites is also mediated by lactate, a metabolite that directly affects MDSCs infiltration in tumor sites. Deletion of the m6A RNA Demethylase Alkbh5 that regulates expression of Mct4, a key enzyme catalyzing rapid transport across the plasma membrane of lactate, inhibits the recruitment of MDSCs and enhances the efficacy of anti–PD-1 treatment (88).

### 4.2 Targeting Expansion of MDSCs

Because MDSCs expansion is known to be regulated by tumor-derived factors, several studies have focused on neutralizing the effects of these factors to improve the efficiency of ICB. Recently, NOD-, LRR-, and pyrin domain-containing protein 3 (NLRP3) has been implicated in causing MDSCs expansion in tumor-bearing mice. In metastatic melanoma, tumor-associated NLRP3/IL-1β signaling induced expansion of MDSCs, leading to reduced NK and CD8+ T-cell activity concomitant with an increased presence of Treg cells in the primary tumors. The combination of NLRP3 inhibition by dapansutrile (OLT1177) and anti-PD-1 treatment significantly increased the antitumor efficacy by reducing MDSCs expansion and limiting MDSCs-mediated T-cell suppression and tumor progression (89). CD8+ T-cell activation in response to PD-1 blockade induced a PD-L1/NLRP3 inflammasome signaling cascade that ultimately led to the recruitment of CXCR2+PMN-MDSCs into tumor tissues through HSP70/TLR4/Wnt5a/CXCL5 signaling axis, thereby dampening the resulting antitumor immune response. The genetic and pharmacologic inhibition of NLRP3 suppressed PMN-MDSCs tumor infiltration and significantly augmented the efficacy of anti-PD-1 antibody immunotherapy in melanoma (90). In murine models, antibody-mediated neutralization of tumor cell-derived IL-1β has been shown to reduce MDSC accumulation, with increased tumor infiltration of CD8+ T cells, resulting in decreased tumor growth and prolonged survival, as well as enhanced responsiveness to anti-PD-1-based therapies in PDAC and renal cancer (91, 92).

IL-18, another downstream target of NLRP3, is also involved in the expansion of MDSCs in the TME. In lymphoma, NLRP3 inflammasome blockade *in vivo* suppressed tumor growth and ameliorated antitumor immunity by decreasing MDSCs, TAMs, and Tregs through the effector cytokine IL-18. Thus, NLRP3 blockers combined with anti-PD-L1 treatment exerted antagonistic effects during lymphoma therapy (116). VEGF, another tumor-derived factor that is involved in promoting MDSCs expansion, might also be a useful target by which to enhance the efficacy of ICB therapy. In malignant mesothelioma (MPM), chemotherapy-mediated suppression of VEGF expression decreased numbers of intratumoral MDSCs and inhibited tumor vessel formation, which significantly enhanced the antitumor effects of anti-PD-1 antibody (117). In melanoma, silencing of VEGF or TGF-β resulted in dramatically delayed tumor growth, associated with decreased Tregs and MDSCs and increased effector T-cell activation in tumor infiltrates, which restored tumor sensitivity to tumor-specific cell therapies and markedly improved the efficacy of anti-PD-1/anti-CTLA-4 treatment (93). Endostar is a novel recombinant human endostatin that exerts its anti-angiogenic effects *via* VEGF-related signaling pathways. Anti-PD-1 combined with endostar reduced MDSCs accumulation and reversed CD8 + T cell suppression through decreasing pro-inflammatory cytokine IL-17 and immunosuppressive factor TGF-β1 levels, which dramatically suppressed tumor growth in Lewis lung carcinoma (LLC) mouse models (94). Finally, a more refined approach uses antibodies recognizing CD200R, a marker expressed on the surface of MDSCs. CD200R+ MDSCs expressed genes involved in cytokine signaling and MDSCs expansion. CD200 expression in the PDAC microenvironment promoted MDSCs expansion and *in vivo* blockade of CD200 can significantly enhance the efficacy of PD-1 checkpoint antibodies compared with single antibody therapies (95).

### 4.3 Promoting MDSCs Differentiation

Another alternative MDSCs‐targeting strategy is to promote the differentiation and maturation of MDSCs. One promising therapeutic target appears to be PGE2, a bioactive lipid metabolite derived from arachidonic acid, which was reported to induce bone marrow stem cells to differentiate into Gr1+CD11b+ MDSCs through its binding to a family of G protein-coupled receptors: E-type prostanoid receptors 1–4 (EP1-4), but either EP2 (AH6809) or EP4 (AH23848) antagonists blocked the induction (118). Chemical inhibition of EP4 by the new EP4 antagonist, TP‐16, significantly decreased the proportion of M-MDSCs, though no significant difference was observed in the proportion of PMN‐MDSCs and decreased the expression of MDSC markers (for both M-MDSCs and PMN‐MDSCs), such as Arg‐1, Ptgs2, IL‐4ra, Ido1, and IL‐10, thus enhancing cytotoxic T‐cell activation and increasing responsiveness to anti-PD-1 therapy in a spontaneous colorectal cancer mouse model (96). MF-766, another potent and highly selective small-molecule inhibitor of the EP4 receptor, synergistically improved the efficacy of anti-PD-1 therapy in CT26 colon adenocarcinoma and EMT6 syngeneic mammary carcinoma mouse models through reduced G-MDSCs, induced M1-like macrophage reprogramming, as well as promoting the infiltration of CD8+ T cells, NK cells, and conventional dendritic cells (cDCs) in the TME (97). Similarly, E7046, an orally bioavailable EP4-specific antagonist, also showed synergistic antitumor activity when combined with anti-CTLA-4 antibodies through impairing tumor-promoting MDSCs differentiation, M2 macrophage polarization, and Tregs-derived immunosuppression (98). The immunomodulatory effect of E7046 is also confirmed in the first-in-human phase I study (119). However, the MDSCs‐targeting and antitumor efficacy of these agents combined with ICB has not yet been widely investigated in clinical trials, and more studies are warranted.

Polyamine blocking therapy (PBT) can redirect the differentiation of MDSCs to pro-inflammatory M1-like macrophages through the downregulated expression of p-STAT3, an oncogenic transcription factor whose activation is implicated in MDSCs differentiation and survival. PBT significantly enhanced the antitumor efficacy of PD-1 blockade in both 4T1 mammary carcinoma and B16F10 melanoma tumors resistant to anti-PD-1 monotherapy, increasing tumor-specific cytotoxic T cells and survival of tumor-bearing animals beyond that with PBT or PD-1 blockade alone (99). Several studies indicate that all-trans retinoic acid (ATRA) is another agent that can promote myeloid cells maturation and reduce the number of MDSCs in the TME. ATRA converts MDSCs into DCs while intervening in the polarization of macrophages. A nano-educator (NE) that when loaded with ATRA and anti-PD-1 antibodies (aPD-1) instructs myeloid cells to assist T cells towards revitalizing anti-PD-1 therapy, broadening the application of aPD-1 in the treatment of anti-PD-1-resistant tumors (100). In a randomized phase II clinical trial treating advanced melanoma patients with Ipilimumab plus ATRA, ATRA significantly decreased the frequency of circulating MDSCs compared to Ipilimumab treatment alone (101). High-mobility group box 1 (HMGB1), a DAMP with pleiotropic functions, is involved in various intracellular (e.g., chromatin remodeling, transcription, autophagy) and extracellular (inflammation, autoimmunity) processes, which has been associated with both protumor and antitumor functions. HMGB1 has been demonstrated to drive MDSCs, promote the development of MDSCs from bone marrow progenitor cells, contributing to their ability to suppress antigen-driven activation of CD4(+) and CD8(+) T cells (120). Targeting HMGB1 results in drastic reductions of monocytic/granulocytic MDSCs and Tregs, a higher M1/M2 ratio of macrophages, as well as increased activation of both DC and plasmacytoid DC (pDC), without affecting the global number of (CD45+) immune cells. As a consequence, blocking HMGB1 improved the efficacy of anti-PD-1 cancer monoimmunotherapy (102).

### 4.4 Targeting the Immunosuppressive Function of MDSCs

Elimination of MDSCs immunosuppressive activity represents the major therapeutic approach to re-establishing T-cell activity and ICB success. MDSCs can be functionally inactivated by targeting their suppressive machinery. One potential approach is to block the signaling pathways that regulate the production of suppressive factors by these cells. The most prominent factors implicated in MDSCs suppressive activity includes arginase, NO, ROS, and PGE2. A large number of preclinical studies have shown that targeting immunosuppressive mediators produced by MDSCs can enhance the efficacy of ICB therapy. One potential target by which this might be achieved is NADPH oxidase (NOX2). NOX2 is required for the production of ROS by MDSCs, thereby inducing their suppressive function. Accordingly, Histamine dihydrochloride (HDC), a NOX2 inhibitor, is found to reduce the ROS formation by intratumoral MDSCs, which improved antitumor T-cell responses and enhanced the antitumor efficacy of PD-1 and PD-1 ligand checkpoint blockade (103). Similarly, the Aurora A inhibitor alisertib is also reported to disrupt the immunosuppressive functions of MDSCs by inhibiting Stat3-mediated ROS production, which triggers the rapid accrual of cytotoxic T cells, and efficiently inhibits the proliferation of tumor cells. As a result, alisertib combined with PD-L1 blockade shows synergistic efficacy in the treatment of mammary tumors (104). However, interestingly, promoting the production of ROS in MDSCs can also enhance the efficacy of ICB. Phenformin induces excessive production of ROS in G-MDSCs, which reaches a toxic threshold level in G-MDSCs and contributes to its deleterious effects on these cells. What is more, phenformin significantly decreased the expression of arginase 1, S100A8, and S100A9 that are critical for immune-suppressive activities of MDSCs. Resultantly, the inhibitory effect of phenformin on G-MDSC-driven immune suppression induces CD8+ T-cell infiltration and improves the antitumor activity of PD-1 blockade immunotherapy in melanoma (105).

Poly (ADP-ribose) polymerase inhibitors (PARPi) are also able to modulate MDSCs suppressive function involving a reduction in Arg-1/iNOS/COX-2. In preclinical colon cancer mouse models, Partial PARPi inhibition with metronomic therapy, such as olaparib and talazoparib, has been demonstrated to reactivate antitumor immunity through increases in intratumoral T-cell function and cytotoxicity, thus enhancing anti-PD-1 immunotherapy (106). Recent clinical trials with olaparib plus durvalumab, a human monoclonal antibody that targets PD-1, have also shown acceptable toxicity and improved patients’ outcome in metastatic castration-resistant prostate cancer (mCRPC) (107).

The selective class I histone deacetylase (HDAC) inhibitor entinostat (ENT) has been reported to have an inhibitory effect on MDSCs immunosuppressive functions and converts ICB treatment resistance in several preclinical tumor models. In breast cancer and metastatic pancreatic cancer, the addition of ENT to checkpoint inhibition led to significantly decreased suppression by G-MDSCs through decreasing Arg-1 production and diminishing availability of the PD-L1/PD-1 T-cell inhibitory pathway and increase in activated granzyme-B-producing CD8+ T effector cells in the TME (108). In lung and renal cell carcinoma, ENT inhibited the immunosuppressive function of both PMN- and M-MDSCs populations by significant reduction in Arg-1, iNOS, and COX-2 levels and hence enhanced the antitumor effect of PD-1 inhibition (109). Recently, the anticonvulsant drug valproic acid (VPA), due to its inhibition of histone deacetylases, was found to attenuate the immunosuppressive function of PMN-MDSCs through significantly downregulating Arg1 and Ptges expression, thus enhancing CD8+ T-cell activation and NK cell proliferation in tumors. Consequently, the combination of VPA plus an anti-PD-1 antibody was more effective than either agent alone in both the lymphoma and melanoma tumor models (110).

Blocking the immunosuppressive function of MDSCs can also be achieved by targeting phosphoinositide 3-kinase (PI3K). P110δ and p110γ isoforms of PI3K, which are expressed primarily in hematopoietic cells, have been implicated in immunosuppression mediated by myeloid cells in solid tumors. Inhibitor of PI3Kδ/γ, such as (S)-(-)-N-[2-(3-Hydroxy-1H-indol-3-yl)-methyl]-acetamide (SNA), (-)-4-O-(4-O-β-D-glucopyranosylcaffeoyl) quinic acid (QA), and IPI-145, reversed the suppressive effects of G-MDSCs on the proliferation of T lymphocytes through significantly reducing the expression of NOS2 and Arg1 transcript levels in G-MDSCs and promoting cytotoxic T-cell-mediated tumor regression, resultantly enhancing the therapeutic efficacy of anti-PD1 treatment in osteosarcoma tumor (111), colon tumor (112), and oral cancer (121), respectively.

Genetic and pharmacologic inhibition of myeloid cell receptor tyrosine kinases TYRO3, AXL, and MERTK have been demonstrated to diminish suppressive enzymatic capabilities of MDSCs and augment anti-PD-1 therapy in melanoma in part through regulation of STAT3 serine phosphorylation and nuclear localization (113). Semaphorin4D, originally characterized for its axonal guidance properties, also modulates global immune cytokine profiles and myeloid cell polarization within the TME. Treatment of tumor-bearing mice with Sema4D mAb reduced PMN-MDSCs suppressive capacity through inhibition of Sema4D-driven ERK- and STAT3-dependent arginase expression and abrogated PMN-MDSCs recruitment through reducing MAPK-dependent chemokine production by tumor cells in murine oral cancer-1 (MOC1) tumors, which led to enhanced tumor infiltration by CD8+ TIL and activation of tumor-draining lymph node T lymphocytes in response to tumor antigen. Sema4D mAb in combination with either CTLA-4 or PD-1 blockade enhanced rejection of tumors or tumor growth delay, resulting in prolonged survival with either treatment (122). MDSCs derived-TGF-β mediated PD-1 high expression on CD8+ T cells, which led to being resistant to PD-1/PD-L1 blockade in the TME. Dual PD-1/PD-L1 and TGF-β signaling pathway blockades synergistically restored the function and antitumor ability of antigen-specific CD8+ T cells *in vitro*/vivo assay in ESCC (123). However, in a cohort study of patients with advanced NSCLC, higher levels of FoxP3+ Treg cells and TGF-β were associated with favorable clinical response to anti-PD-1 immunotherapy (124).

Recently, fatty acid transport protein 2 (FATP2), overexpressed in PMN-MDSCs, has been demonstrated to be a promising target to reduce MDSCs immunosuppressive functions. FATP2-mediated suppressive activity of G-MDSCs involved the uptake of arachidonic acid and the synthesis of PGE2. The selective pharmacological inhibition of FATP2 by lipofermata abrogated the activity of PMN-MDSCs and substantially delayed tumor progression. Resultantly, the combination of CTLA4 antibody with lipofermata in LLC-bearing mice caused a potent antitumor effect, where the administration of each monotherapy failed to block tumor progression (125). Additionally, lipofermata inhibition of FATP2 in MDSCs was also found to decrease lipid accumulation-induced ROS, block immunosuppressive activity, and consequently enhance anti-PD-L1 tumor immunotherapy *via* the activation of T cells in melanoma and lung cancer (126).

Evidence suggests that there is a broad range of methods that will be effective for targeting the number and/or function of MDSCs *in vivo*. These strategies will undoubtedly help to expedite clinical applications to enhance the efficacy of ICB therapy.

### 4.5 Targeting Tumor-Derived Vesicles

Extracellular vesicles (EVs), membrane-bound nanoparticles ranging from 40 to 1,000 nM and released ubiquitously by both normal cells and tumors, are thought to be a critical means of cell-cell communication. It is generally agreed that they carry proteins, lipids, and genetic material, such as RNAs and microRNAs (miRs), that transmit an array of signals to target cells (127, 128). Recent data have suggested that tumor-derived EVs have been implicated in the induction of MDSCs in many cancers. Immunosuppressive monocytes in glioblastoma (GBM) are an obstacle to effective immunotherapy. Studies have shown that GBM EVs could induce immunosuppressive monocytes, including MDSCs and non-classical monocytes (NCMs), rather than direct T-cell inhibition to promote immunosuppression (129). Melanoma EVs are also reported to generate immunosuppressive MDSCs by upregulating PD-L1 *via* TLR4 signaling (130). A set of microRNAs contained in melanoma-derived EVs is found to be responsible for the conversion of monocytes into MDSCs and their baseline levels in plasma clustered with the clinical efficacy of CTLA-4 or PD-1 blockade in melanoma patients (1). Acute myeloid leukemia (AML) EVs are taken-up myeloid progenitor cells and thereby induce proliferation in the target MDSC population through the high expression of c-myc drived by MUC1 oncoprotein (131). Additionally, palmitoylated proteins on AML-derived EVs have been demonstrated to promote MDSC differentiation *via* TLR2/Akt/mTOR signaling (132). Colorectal cancer MC38 cell-derived EVs are involved in the increase of PD-L1+ M2-like macrophage, Treg cell, and MDSC populations, resulting in the establishment of the immunosuppressive tumor microenvironment (133). Small extracellular vesicles (sEVs) derived from the hypoxic tumor microenvironment of head and neck squamous cell carcinoma (HNSCC) recruited MDSCs to form a premetastatic niche by delivering lysyl oxidase like 2 (LOXL2) to fibroblasts to induce fibronectin production (134). Breast cancer-derived exosomal microRNA-200b-3p recruits MDSCs and promotes specific lung metastasis through regulating CCL2 expression in lung epithelial cells (135).

## 6 Conclusions

Currently, ICB therapy presents significant limitations in the treatment of cancer patients, although there are already promising results in some cancer types such as non-small-cell lung cancer and melanoma. The limited benefit in patients with other solid malignancies is a major obstacle associated with ICB therapies. MDSCs, as one of the main promoters of cancer, provide comfortable conditions for tumor growth and implicate in resistance of ICB therapy by exerting potent immunosuppressive functions. The combination of double blockade with drugs that function on MDSCs plus ICB may come to play a key role in the treatment of different solid neoplasms. Additionally, the current research on targeting MDSCs to enhance ICB therapy mainly focuses on reversing the immunosuppressive state of the tumor microenvironment. However, the tumor-promoting effect of MDSCs is not limited to inhibiting antitumor immune response; it can also promote tumor angiogenesis and induce cancer stem cells, which provide more evidence to elucidate the role of a double blockade. Further preclinical and clinical studies will be required to assess the safety and efficacy of these combination therapies

## Author Contributions

XL contributed to the manuscript writing and revision, paper gathering and information analysis, and providing initial ideas. XL, JZ, XD, XG, YL, XH, JL, and CW contributed to providing revision advice and checking. All authors contributed to the article and approved the submitted version.

## Funding

The authors’ research was funded by grants from the National Natural Science Foundation for Key Programs of China (81774261, CW) and Project of Administration of Traditional Chinese Medicine of Guangdong Province of China (20191007, XL).

## Conflict of Interest

The authors declare that the research was conducted in the absence of any commercial or financial relationships that could be construed as a potential conflict of interest.

## Publisher’s Note

All claims expressed in this article are solely those of the authors and do not necessarily represent those of their affiliated organizations, or those of the publisher, the editors and the reviewers. Any product that may be evaluated in this article, or claim that may be made by its manufacturer, is not guaranteed or endorsed by the publisher.
